# Suppression (but Not Reappraisal) Impairs Subsequent Error Detection: An ERP Study of Emotion Regulation's Resource-Depleting Effect

**DOI:** 10.1371/journal.pone.0096339

**Published:** 2014-04-28

**Authors:** Yan Wang, Lixia Yang, Yan Wang

**Affiliations:** School of Psychology and Cognitive Science, East China Normal University, Shanghai, China; Erasmus University Rotterdam, Netherlands

## Abstract

Past event-related potentials (ERPs) research shows that, after exerting effortful emotion inhibition, the neural correlates of performance monitoring (e.g. error-related negativity) were weakened. An undetermined issue is whether all forms of emotion regulation uniformly impair later performance monitoring. The present study compared the cognitive consequences of two emotion regulation strategies, namely suppression and reappraisal. Participants were instructed to suppress their emotions while watching a sad movie, or to adopt a neutral and objective attitude toward the movie, or to just watch the movie carefully. Then after a mood scale, all participants completed an ostensibly unrelated Stroop task, during which ERPs (i.e. error-related negativity (ERN), post-error positivity (Pe) and N450) were obtained. Reappraisal group successfully decreased their sad emotion, relative to the other two groups. Compared with participants in the control group and the reappraisal group, those who suppressed their emotions during the sad movie showed reduced ERN after error commission. Participants in the suppression group also made more errors in incongruent Stroop trials than the other two groups. There were no significant main effects or interactions of group for reaction time, Pe and N450. Results suggest that reappraisal is both more effective and less resource-depleting than suppression.

## Introduction

### Emotion regulation

Emotion regulation refers to the automatic or controlled processes that people use to influence the nature and strength of the emotions and how emotions are experienced and expressed [1]. According to the process model of emotion regulation, there are two classes of emotion regulation approaches, the antecedent-focused strategies and the response-focused strategies [1]. Those that occur prior to an emotion response are antecedent-focused, such as situation selection, situation modification, attentional deployment, and cognitive change. Those that occur after an emotion response is generated are response-focused, such as response modulation. Most studies of antecedent-focused emotion regulation processes focused on the effects of attentional deployment (directing attention toward or away from particular aspects of the situation) and cognitive change (changing the interpretation of a situation so as to alter its emotional impact). On the other hand, response modulation, a response-focused process, typically refers to the efforts to suppress the expression or experience of emotion.

Abundant studies explored the effect of different emotion regulation strategies (distraction, concentration, suppression, reappraisal, etc.) on emotion experiences and related outcomes [2]. Evidence showed that: reappraisal is one of the most effective strategies for emotion regulation; distraction was an effective way to regulate emotions; the effect of suppression on emotions varied from zero to small; etc. When comparing different emotion regulation strategies, one question is which one most successfully reduces or amplifies the emotion outcome, and another question is which one has the least cost [3]. Although numerous studies addressed the effectiveness of different strategies, an unresolved question is the cognitive and physiological consequences or costs of emotion regulation [2].

### Emotion regulation and depletion of self-control resources

According to strength model of ego-depletion, coping with stress, regulating negative emotions, and resisting temptations require self-control [4]–[5]. Engaging in self-control (such as controlling thoughts, regulating emotions, overcoming unwanted impulses, controlling attention) will lead to impaired task performance on subsequent self-control tasks (impulse control, choice and volition, cognitive processing, social processing, etc.), know as ego-depletion [6]. The major idea is that self-control is a limited resource that gets depleted after exertion, resulting in reduced capacity for further self-control. The regulation of emotion has been shown to drain self-control resources and is a common means to invoke ego depletion in the dual-task paradigm [6]. This is because regulating emotion requires an individual to override the innate tendency to display emotions in response to environmental stimuli.

In an event-related brain potential (ERP) study, subjects watch an upsetting movie, and half subjects were required to suppress their emotion reactions to the movie, half subjects simply watched the movie carefully [7]. Then, all subjects completed an ostensibly unrelated task, the color-naming Stroop task. Throughout the experiment, participants' brain electrical activities were recorded, and the error-related negativity (ERN) during the Stroop task was analyzed. Results showed that after exerting emotion suppression, the participants performed worse on the Stroop task. Furthermore, relative to the control group, subjects in the emotion-suppression group exhibited reduced ERN amplitudes when making errors on the Stroop task. ERN is an evoked negative potential thought to originate from anterior cingulate cortex (ACC) [8] and theorized to reflect error detection [9]. Thereby, emotion suppression seemed to weaken the neural systems for monitoring errors and conflicts (e.g. ACC). However, a study using fMRI reached a different conclusion [10]. After suppressing emotions during a picture-viewing task, participants completed a Stroop task, and fMRI images were acquired during both the initial (emotion suppression) and subsequent self-control task (Stroop task). Results showed that two brain areas were activated during both the emotion suppression task and the Stroop task, the right lateral prefrontal cortex (LPFC) and the medial frontal cortex (MFC, including the dorsal part of ACC). However, only LPFC showed reduced activation after emotion suppression during the Stroop task, and MFC did not show a similar pattern of reduced activity. The authors concluded that LPFC was sensitive to the aftereffects of self-control exertion and MFC was not sensitive as such [10].

### Do different emotion regulation strategies uniformly consume self-control resources?

Studies inspired by ego depletion theory and process model of emotion regulation have an interesting crossover, namely the influence of emotion regulation on cognitive performance. In the emotion regulation line of research, results showed that expressive suppression (which consists of concealing outward signs of emotion) leads to poor memory for the emotional stimuli [11]. Reappraisal (which involves changing an emotional situation's meaning in a way that alters its emotional impact) is more effective than suppression in decreasing emotion experience and behavioral expression. Besides, reappraisal has less physiological or cognitive costs (such as impairing memory) than suppression [12]. Reappraisal may even prime cognitive resources. An ERP study showed that increasing negative emotions by reappraisal enhanced subsequent cognitive control, as reflected in reduced Stroop interference and enhanced Stroop-locked sustained potential interference effect [13]. On the other hand, decreasing negative emotions through reappraisal had no effect on subsequent Stroop performance or Stroop-related ERPs. However, a recent study revealed that reappraising and suppressing emotion experience to unpleasant pictures both slowed down reactions during a concurrent auditory discrimination task [14]. In the ego depletion line of research, one of the mostly used techniques for exhausting self-control resources is having participants engage in an emotional suppression task [6]. Recent result showed that participants who suppressed their emotions performed worse in subsequent stop signal task than those who accepted their emotions [15]. Furthermore, EEG and fMRI research suggests that suppression carry over costs on subsequent cognitive control task which is evident in the neural level, although there are controversies concerning whether ACC activities would be reduced after emotion suppression [7], [10].

### Overview and hypotheses

According to ego-depletion studies, effortful acts of self-regulation (such as emotion regulation) drew on the limited resources, thus temporarily deplete people's capacity to further regulate in seemingly unrelated domains (such as cognitive control). Do different forms of emotion regulation equally cognitively effortful and resource-depleting? Might some emotion regulation strategies even enhance subsequent cognitive control? Considering existing theories and evidence (although controversies), we predicted some emotion regulation strategies (e.g. suppression) were more resource-consuming than others (e.g. reappraisal). To test this prediction, we trace the Stroop performance after initial emotion regulation. Furthermore, we examine the ERP components of performance monitoring cognitive control functions, namely the error-related negativity (ERN), post-error positivity (Pe) and N450 [9], [16]. They reflect early error detection, error awareness, and conflict monitoring, respectively. Combining behavioral and electrophysiological measures, we can compare various emotion regulation strategies' diversified impact on subsequent cognitive control.

In this study, we focus on two major emotion regulation strategies, reappraisal and suppression. We combine behavioral and ERP measures to explore emotion regulation's influences on subsequent cognitive control during a Stroop task. To our knowledge, no research hitherto directly compares emotion suppression and reappraisal's differential impact on subsequent cognitive control at the electrophysiological level. We hypothesized: first, compared to emotion suppression, reappraisal was more successful at reducing the movie-elicited emotions; second, suppression group performed worse during the Stroop task relative to the control group, both at the behavioral and the electrophysiological level; third, compared to emotion suppression, reappraisal was less destructive on subsequent cognitive control task, including one or more of the performance monitoring ERP components during the task.

## Methods

### Ethics statements

This study was approved by the ethics committee of East China Normal University. All participants provided written informed consent.

### Participants

Fixty-eight Chinese university students (mean age 22.27 years) participated in the experiment for monetary compensation (¥70, approximately US$12), and all participants were native Chinese speakers. All participants were healthy, right-handed, with normal or corrected-to-normal vision, and reported no history of neurological or psychiatric disorders. Data from eleven subjects were excluded because of excessive error rates (>30%) on incongruent Stroop trials (n = 4), excessive EEG artifacts (all error trials were removed after artifact rejection, n = 4), or fewer than six error trials after artifact rejection (n = 3) [17]. Participants were randomly assigned to one of three groups: control (n = 15, mean age 23.07 years, 8 women), suppression (n = 16, mean age 22.50 years, 8 women), and reappraisal (n = 16, mean age 21.25 years, 7 women).

### Procedure

After signing an informed consent form, participants completed a questionnaire consisting of demographic information. Then they were fitted with an electrode cap for EEG recording. The experiment was administered individually.

The first part of the experiment (video watching) was explained as a personality and emotion study. All participants were told that they were to watch a film clip, and then they received instructions for the clip. Participants in the suppression group were instructed to closely watch the clip but to suppress both the experience and expression of emotions while watching, and “control your internal reactions to the film and adopt a neutral facial expression” (instructions were adapted from [18]). Participants in the reappraisal group were instructed to closely watch the clip but to “adopt a neutral attitude toward the film contents”, and “think the film objectively and analytically” (instructions adapted from [19]). Participants in the control group were instructed to closely watch the clip. Immediately after watching the clip, participants completed a 16-item state mood questionnaire, followed by two manipulation check items.

The second part of the experiment (Stroop task) was posed as a response time study unrelated to the first study. The Stroop task lasted about 20 minutes, during which continuous EEG activities were recorded. After that, participants rated the Stroop task on two items (difficulty and motivation), and then were asked what they thought the experiment was about. Then they were debriefed, thanked, and dismissed.

### Materials

#### Film clip

A nine-minute excerpt from the movie The Champ, in which a boy witnessed his father died after suffering a severe beating in the ring, was used. This film clip elicits emotion reports of sadness with little other emotion [20].

#### Mood Questionnaire

The Brief Mood Introspection Scale (BMIS) [21], a 16-item instrument that is used to measure mood valence and arousal. The items include calm, content, happy, nervous, sad, etc. Participants indicated how well each adjective or phrase describes their present mood (on a scale from 1 =  definitely do not feel to 4 =  definitely feel). Cronbach's α was 0.725 for valence and 0.684 for arousal.

#### Manipulation check

To determine whether participants followed instructions, we presented subjects with two items: “During the film, I tried not to feel anything at all’’ and “During the film, I reacted completely spontaneously’’ (reverse coded, α = 0.780). Participants indicated their agreement with these statements on a 7-point Likert scale.

#### Stroop task

Stimuli consisted of a series of color words (red, green, blue or yellow in Chinese), each of which was presented in a color that either matched (congruent) or did not match (incongruent) the semantic meaning of the word. Participants were instructed to identify the color in which each word was presented by pressing the corresponding colored key on a keyboard. All stimuli were presented centrally against a black background. Each trial began with a fixation cross (“+”) for 500 ms, followed by a stimulus word presented for 200 ms. Participants were given 1000 ms in which to respond. The inter-trial intervals varied randomly between 1000 ms and 1200 ms. The task contained 5 blocks, each consisting of 72 congruent trials (e.g., the word “red” in red ink) and 36 incongruent trials (e.g., the word “red” in green ink). The mean reaction time (RT) for each trial type was calculated with correct responses only. Stroop interference scores were calculated by subtracting mean RTs associated with congruent trials from mean RTs associated with incongruent trials.

#### Stroop task rating

Participants rated how difficult the “response time” task was (from 1 =  very easy to 7 =  very difficult). Participants also indicated their degree of motivation during the task (from 1 =  not at all to 7 =  very much).

### Electrophysiological Recording and Processing

The EEG signals were recorded through DC- amplifier system and the software “Vision Recorder” (Brain Products, Munich, Germany) from 32 Ag/AgCl electrodes mounted in an elastic cap. Six additional electrodes were attached; to the left and right mastoids serving as reference sites, two outer canthi of the eyes to measure horizontal eye movements (HEOGs), infraoribital, and supraorbital regions of the left eye to measure vertical eye movements and eye blinks (VEOGs). Furthermore, two additional scalp electrodes were used to serve as reference and ground electrodes. Electrode impedances were kept below 5 kΩ. Online signals were recorded from 0.01 to 100 Hz. All signals were digitized with a sample rate of 512 Hz and 24-bit A/D conversion.

EEG data were analyzed by “Vision Analyzer” software (Brain Products, Munich, Germany). Offline, a mathematically linked mastoid reference was applied and EEG and EOG activity was filtered with a band-pass of 0.10–30 Hz (phase shift-free Butterworth filters; 24 dB/octave slope). Eye movement and blink artifacts were corrected using the algorithm developed by Gratton et al. [22]. For the response-locked ERN and Pe, correct and incorrect trials were averaged separately with an epoch from 400 ms pre-response to 800 ms post-response. For the stimulus-locked N450, correct congruent and correct incongruent trials were averaged separately with an epoch from 200 ms pre-stimulus to 1000 ms post-stimulus. An artifact rejection excluded all epochs containing a voltage step of more than 50μV between sample points, a voltage difference of 300μV within a segment, and a maximum voltage difference of less than 0.50μV within 100 ms intervals. A 200 ms time window from 400 to 200 ms before the response was used as the baseline for ERN and Pe. A 200 ms time window from 200 to 0 ms before the stimulus was used as baseline for N450.

Error-trial and correct-trial ERN amplitudes were extracted as the mean amplitude from 20 ms before response and 50 ms after response at FCz [23]. Error-trial and correct-trial Pe amplitudes were calculated as the mean amplitude from 220 to 400 ms after response at Pz [23]. Congruent-trial and incongruent trial N450 amplitudes were calculated as the mean amplitudes for the time interval between 420 and 550 ms after stimulus across FCz, Cz, and CPz [24]. Response-locked ERPs contained an average of 22 error trials and 470 correct trials for control group, 28 error trials and 485 correct trials for suppression group, and 27 error trials and 486 correct trials for reappraisal group. Stimulus-locked ERPs contained an average of 123 incongruent trials and 283 congruent trials for control group, 139 incongruent trials and 303 congruent trials for suppression group, and 121 incongruent trials and 270 congruent trials for reappraisal group. No between group differences were shown for the number of trials retained for averaging (all Fs <1.3, all ps >0.28).

## Results

### Manipulation checks and mood

Descriptive statistics of the main variables were presented in Table 1. We examined responses to the two items to determine whether participants in the suppression group and reappraisal group attempted to regulate their emotions as instructed. One-way analysis of variance (ANOVA) reveal a significant effect of group, F(2, 44)  = 18.280, p<0.001, partial η^2^  = 0.454. Pairwise comparisons showed that participants in the control group (2.800) reported less regulation than the suppression group (4.844, p<0.001) and reappraisal group (5.031, p<0.001), and the last two groups didn't differ (p = 0.642).

**Table 1:** Means and Standard Deviations in parenthesis for Main Variables by group.

	Control	Suppression	Reappraisal
**Manipulation check**			
** Regulating emotion**	2.80(0.96)_a_	4.84(1.34)_b_	5.03(1.06)_b_
**Mood**			
** Sad**	3.33(0.62)_a_	3.25(0.58)_a_	2.63(0.72)_b_
**Stroop task**			
** Accuracy congruent (%)**	94.86(3.61)	94.14(3.79)	94.88(3.92)
** Accuracy incongruent (%)**	91.10(4.57)_a_	87.46(5.56)_b_	91.60(4.46)_a_
** RT congruent (ms)**	575(49.68)	552(53.93)	545(49.93)
** RT incongruent (ms)**	624(53.44)	605(64.65)	592(65.98)
**ERP amplitude**			
** ERN (μV)**	−6.26(4.77)_a_	−1.93(4.08)_b_	−5.61(5.85)_a_
** CRN (μV)**	3.80(3.86)	3.37(3.49)	4.20(5.10)
** ΔERN (μV)**	−10.07(6.27)_a_	−5.27(4.68)_b_	−9.81(5.94)_a_
** Pe (μV)**	4.77(5.29)	6.50(5.23)	8.58(6.13)
** Correct Positivity (μV)**	0.62(4.75)	0.07(4.76)	1.22(4.63)
** ΔPe (μV)**	4.14(4.26)	6.42(6.13)	7.36(5.21)
** N450 incongruent (μV)**	4.00(5.91)	6.14(4.85)	5.13(3.39)
** N450 congruent (μV)**	4.98(5.28)	8.11(5.19)	6.71(2.63)
** ΔN450(μV)**	−0.97(1.43)	−1.97(1.77)	−1.58(1.53)

Note. Row means that do not share a subscript are significantly different, p<0.05.

One-way ANOVA on the BMIS valence subscale showed that the three group did not differ in the mood valence after watching the movie clip, F(2, 44)  = 0.162, p = 0.851, partial η^2^  = 0.007. One-way ANOVA on the BMIS arousal subscale showed a marginally significant effect of group, F(2, 44)  = 3.088, p = 0.056, partial η^2^  = 0.123. Pairwise comparisons indicated that participants in the reappraisal group showed lower arousal level than the control group (p = 0.034) and the suppression group (p = 0.042), and the last two groups didn't differ (p>0.999). Previous study showed that the film clip we used here elicits sad emotion [20], so we further analyzed the “sad” item in the BMIS with one-way ANOVA. Results showed a significant effect of group, F(2, 44)  = 5.749, p = 0.006, partial η^2^  = 0.207. Pairwise comparisons indicated that participants in the reappraisal group reported less sadness than the control group (p = 0.004) and the suppression group (p = 0.008), and the last two groups didn't differ (p = 0.719).

After the Stroop task, participants in three groups indicated that they found the Stroop task equally difficult, F(2, 44)  = 1.105, p = 0.340, partial η^2^  = 0.048. In addition, they did not differ on the motivation to complete the Stroop task, F(2, 44)  = 2.702, p = 0.081, partial η^2^  = 0.121.

### Stroop Performance

We calculated the percentage of correct responses (accuracy) separately for congruent and incongruent trials. The Stroop accuracy were then analyzed using a 3 (group: control vs. suppression vs. reappraisal) × 2 (trial type: congruent vs. incongruent) mixed-model analysis of variance (ANOVA), with the last factor repeated. This analysis revealed a significant effect for trial type, F(1, 44)  = 70.066, p<0.001, partial η^2^  = 0.614, suggesting participants made fewer errors on congruent than on incongruent trials. The main effect of group was not significant, F(2, 44)  = 1.821, p = 0.174, partial η^2^  = 0.076. The interaction between trial type and group was significant, F(2, 44)  = 3.849, p = 0.029, partial η^2^  = 0.149. Follow-up analysis showed that participants in the suppression group made more errors (12.5%) in incongruent trials as compared to participants in the control group (8.9%, p = 0.045) and reappraisal group (8.4%, p = 0.021), and the latter two groups did not diff (p = 0.777). The error rates for congruent trials did not differ among the three groups (5.1%, 5.8%, and 5.1% for control, suppression, and reappraisal group, respectively), all ps >0.5.

The Stroop RTs for correct response only were analyzed using a 3 (group: control vs. suppression vs. reappraisal) × 2 (trial type: congruent vs. incongruent) mixed-model analysis of variance (ANOVA), with the last factor repeated. This analysis revealed a significant effect for trial type, F(1, 44)  = 276.190, p<0.001, partial η^2^  = 0.863, suggesting participants responded more quickly on congruent than on incongruent trials (the typical Stroop interference effect). The main effect of group (F(2, 44)  = 1.273, p = 0.290, partial η^2^  = 0.055) and the interaction effect (F(2, 44)  = 0.377, p = 0.688, partial η^2^  = 0.017) were both insignificant. One-way ANOVA on the Stroop interference scores showed that the group effect was not significant, F(2, 44)  = 0.377, p = 0.688, partial η^2^  = 0.017.

### ERN

**Figure 1:**
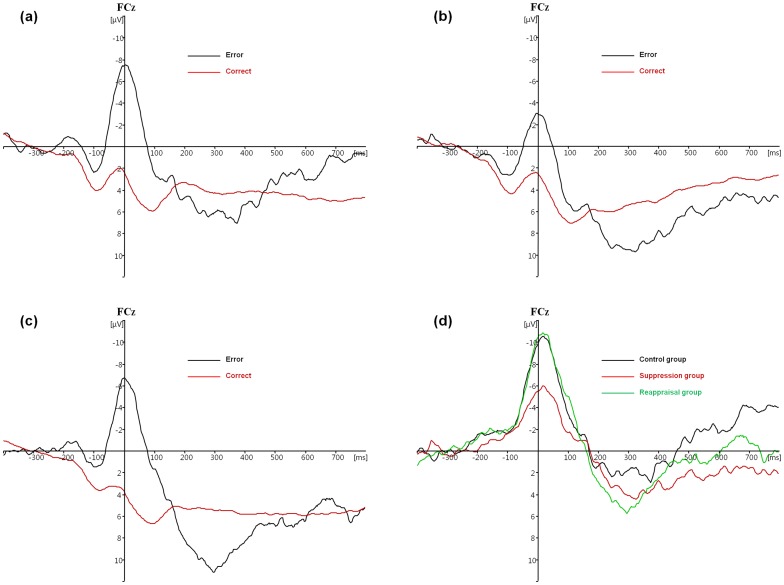
Response-locked ERN at electrode FCz in the (a) control and (b) suppression and (c) reappraisal groups for correct versus error trials, and (d) the average difference waveform (error trials minus correct trials) in each group. Zero in the X-axis indicates the time of key press.

A 3 (group: control vs. suppression vs. reappraisal) × 2 (response type: error vs. correct) mixed-factor ANOVA on ERN amplitude with the last factor repeated was performed. A significant main effect of response was found, F(1, 44)  = 104.786, p<0.001, partial η^2^  = 0.704, with error responses generating larger negative reflections than correct responses. The main effect of group was not significant, F(2, 44)  = 1.200, p = 0.311, partial η^2^  = 0.052. The interaction between group and response type was significant, F(2, 44)  = 3.600, p = 0.036, partial η^2^  = 0.141. That is, although the three groups showed similar amplitudes on correct trials (i.e., the CRN; F(2, 44)  = 0.155, p = 0.857, partial η^2^  = 0.008), the effect of group on the ERN amplitudes was significant, F(2, 44)  = 3.495, p = 0.039, partial η^2^  = 0.137. Specifically, the suppression group displayed a smaller ERN (M = −1.93μV) than the control group (M = −6.26μV, p = 0.019) and the reappraisal group (M = −5.61μV, p = 0.042), with no significant ERN difference between the control and reappraisal group (p = 0.716). Taken another way, although all three groups showed larger negative amplitudes on error trials than on correct trials (i.e., ERN > CRN, all ps <0.001), the ERN versus CRN difference wave (ΔERN) amplitudes differed among the three groups, F(2, 44)  = 3.630, p = 0.035, partial η^2^  = 0.142. The ΔERN in the suppression group (M = −5.27) was smaller than the control group (M = −10.07, p = 0.022) and the reappraisal group (M = −9.81, p = 0.027), and ΔERN of the control and reappraisal group did not differ (p = 0.900) (see Figure 1).

The suppression group made more errors on incongruent trials than the other two groups, which might cause the ERN difference between the suppression group and the other two groups. To test this possibility, we analyzed thirty-three subjects (eleven for each group) matched for their error rate on incongruent trials (9.9% for control group, 10.2% for suppression group and 9.8% for reappraisal group). One-way ANOVA on ERN showed a marginally significant effect of group, F(2, 30)  = 0.071, partial η^2^  = 0.168, with suppression group showing smaller ERN than the other group. Therefore, the ERN amplitude difference among the three groups could not be fully explained by difference on error rate.

### Pe

**Figure 2:**
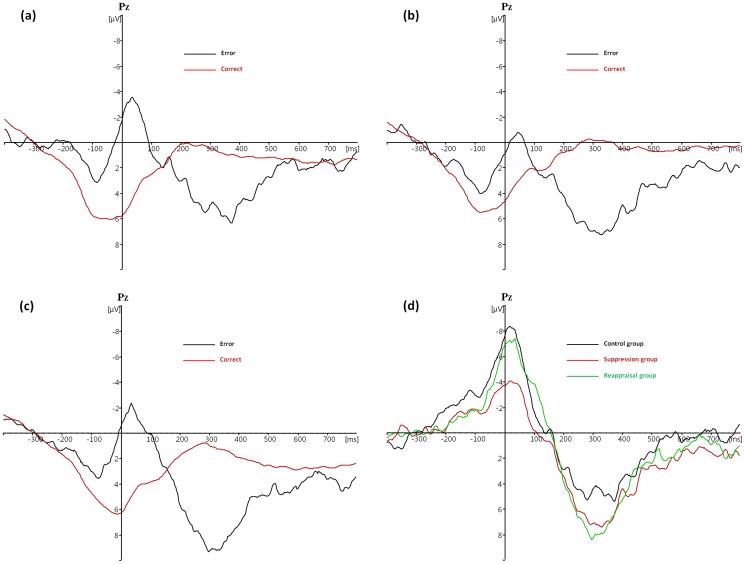
Response-locked Pe at electrode Pz in the (a) control and (b) suppression and (c) reappraisal groups for correct versus error trials, and (d) the average difference waveform (error trials minus correct trials) in each group. Zero in the X-axis indicates the time of key press.

A group (control vs. suppression vs. reappraisal) × response type (error vs. correct) mixed-factor ANOVA for Pe amplitude revealed a significant main effect of response type, with lager Pe amplitude for error trials compared to correct trials, F(1, 44)  = 60.218, p<0.001, partial η^2^  = 0.578. The main effect of group was non-significant, F(2, 44)  = 1.305, p = 0.364, partial η^2^  = 0.045. The group × response type interaction was not significant, F(2, 44)  = 1.508, p = 0.232, partial η^2^  = 0.064, suggesting that the ΔPe among the three groups did not differ (see Figure 2).

### N450

**Figure 3:**
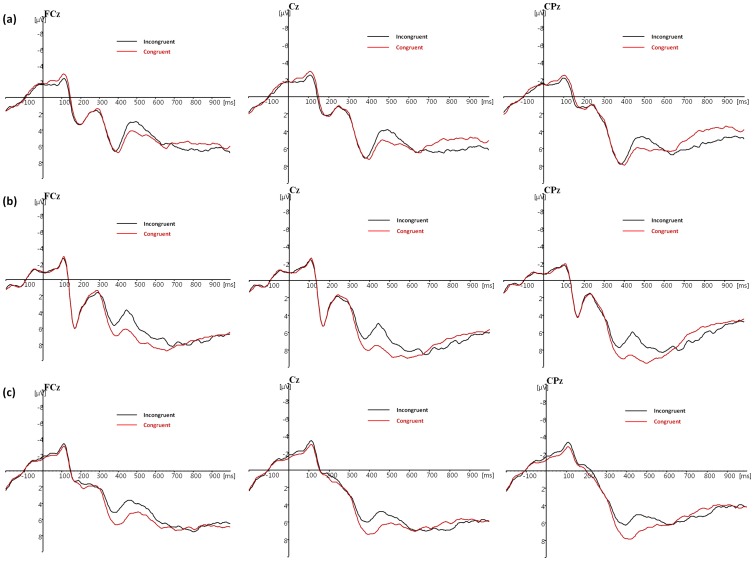
Stimulus-locked N450 at electrode FCz, Cz, and CPz in the (a) control and (b) suppression and (c) reappraisal groups for incongruent versus congruent trials. Zero in the X-axis indicates the time of stimulus onset.

The group (control vs. suppression vs. reappraisal) × trial type (congruent vs. incongruent) mixed-factor ANOVA showed a significant main effect of trial type, with more negative N450 amplitude on incongruent trials relative to congruent trials, F(1, 44)  = 42.263, p<0.001, partial η^2^  = 0.490 (see Figure 3). The main effect of group was non-significant, F(2, 44)  = 1.279, p = 0.289, partial η^2^  = 0.055. The group×trial type interaction was not significant, F(2, 44)  = 1.530, p = 0.228, partial η^2^  = 0.065.

## Discussion

The present study aimed to replicate and extend previous findings indicating that emotion regulation may influence subsequent cognitive control [7], [13], as reflected in behavioral and electrophysiological indices. We examined the impact of suppression and reappraisal on subsequent Stroop performance and on three performance monitoring-related ERP components: the ERN, Pe, and N450 [9]. The results showed that emotion suppression during a sad movie clip led to attenuated error detection during a subsequent Stroop task, as indexed by ERNs. However, reappraisal didn't weaken subsequent error detection, with ERN amplitude corresponding to a solely watching control group. Suppressing also led to more errors during incongruent Stroop trials, as compare to the equivalent errors of reappraisal group and control group. As to the effect of regulating emotion, reappraisal is more successful than suppression, reflected in lower arousal and sadness. The results suggested that adopting a neutral and objective attitude toward a sad movie could effectively decrease sadness without impairing subsequent error detection in a Stroop task. However, suppressing the experience and expression of emotions during the movie didn't reduce the sad emotion, yet impaired subsequent error detection in the Stroop task. No significant group differences were demonstrated for reaction time and the other two ERP components.

Extant ego-depletion literature revealed that regulation of emotion consumed self-control resources and led to reduced capacity for further self-control (including cognitive control) [6]. More recent studies explored the neural mechanisms that give rise to ego-depletion [25]. Neuroscientists have posited that cognitive control relies on two separate neural systems, the error-detection system and the regulatory system [26], [27]. The former is sensitive to the discrepancies between the intended and actual responses, and is supported by ACC. The latter receives the discrepancy information and carries out the desired responses while inhibiting the inappropriate responses, and is supported by the prefrontal cortex (PFC). Neuro-imaging studies have suggested that brain regions involved in cognitive control (the PFC and the ACC) are also activated during emotional regulation [28], self-control resources depletion (e.g. after attention control) leads to a failure to recruit top-down prefrontal regions involved in emotion regulation [29] and impulse control [30]. Therefore, the reason that emotion regulation impairs subsequent cognitive control might be that it drains error detection and/or regulatory neural systems. Furthermore, the type of regulation one employs (e.g. suppression versus reappraisal) may determine whether cognitive resources are drained or even primed.

In the current study, we investigated the impact of regulating emotion by two different strategies on subsequent cognitive control. Our results suggested that suppression taxed the error detection system more than reappraising the emotion-eliciting situation, as reflected in reduced ERN amplitude during Stroop task. Although both ERN and Pe are error-related, the current study found that emotion regulation did not alter Pe. Past studies suggested that ERN reflected early error detection and might not signal a consciously perceived error, yet Pe implied error awareness [31]. Emotion suppression had aftereffects on accuracy during incongruent trials and ERN amplitude, although these two effects seemed to be dissociable. Reinforcement-learning and conflict-monitoring accounts of ERN suggest that the amplitude of ERN is related to performance measures and it is used to improve subsequent performance [32]. However, there are studies showing dissociations between ERN and behavioral measures, suggesting that motivational factors might moderate ERN amplitude [33], [34]. Besides limited resources model, an alternative explanation for ego-depletion effect was reduced motivation [6]. These two accounts are not irreconcilable. The higher error rate and weaker ERN in the suppression group might be caused by decreased resources and reduced motivation, although this possibility need to be further replicated and clarified. Our study proved that ERN amplitude was reduced after emotion suppression, which was similar to the findings of an earlier ERP study [7]. We extended this study by comparing the resource-depleting aftereffects of two different emotion regulation strategies, and revealed their differential impact on ERN. On the other hand, in the current study, reappraisal did not alter the Stroop performance and the error detection system. Previous finding suggested that increasing negative emotions by reappraisal enhanced subsequent cognitive control, whereas decreasing negative emotions by reappraisal has no effect on Stroop performance or Stroop-related ERPs [13]. In our study, we instructed subjects to adopt a neutral attitude as they watch the sad film, which suggested them to decrease negative emotion. Taken together, results seem to support the notion that decrease negative emotion by reappraisal did not affect electrophysiological indices of performance monitoring.

An fMRI study explored the aftereffects of emotion suppression on subsequent Stroop task, and revealed that the right LPFC showed reduced activity during Stroop task after emotion suppression [10]. Although both the MFC and the LPFC showed overlapping activation between emotion suppression and Stroop task, MFC (including ACC) failed to show the similar pattern of reduced activation during Stroop task. These results seemed inconsistent with EEG studies using ERN as a marker for ACC activity, which showed that ERN was reduced after emotion suppression [7, and ours]. Although ACC may be the neural generator of the ERN, other areas such as supplementary motor area (SMA) and pre-SMA were also candidate sources of ERN [32]. Furthermore, connectivity between ACC and the LPFC appears to be critical for generating the ERN. Due to the methodological difference between fMRI and EEG, we refrained from directly comparing the fMRI findings with ours. Besides, there exists another method difference. The emotion suppression fMRI study used a picture viewing task, and the EEG studies used a video viewing task. Suppressing emotions during pictures versus video may tax the neural systems differently [28].

Our findings have implications for emotion regulation theory. Recent emotion regulation studies compared the cognitive costs of different strategies (e.g., suppression, reappraisal, distraction), utilizing a resource-consuming perspective [3], [14], [15]. These studies explored the effect of regulating emotion on concurrent or subsequent cognitive tasks, such as auditory discrimination, stop signal, and Stroop task. Our study extended these behavioral studies and revealed that different emotion regulation strategies can diversely impact error-related brain activities during subsequent unrelated Stroop task. Suppression and reappraisal are among the mostly studied emotion regulation strategies, yet each of them was often defined and operationalized differently by different researchers [2]. Emotion suppression may refer to suppressing the expression of emotion, suppressing the experience of emotion, or suppressing both expression and experience. We used the last definition, for the purpose of comparing our results with previous ego-depletion studies [7]. A limitation of this approach is that we can't say for sure which component (expression or experience suppression) is more harmful for later error detection, which should be clarified in future research. Differences in how reappraisal is operationalized also exist. Some studies asked participants to reinterpret the emotional stimuli, others instructed participants to adopt a neutral and objective perspective so as to distancing themselves from the emotional stimuli, and still others told participants to reappraise the emotional response [2], [35]. Studies suggested that not all forms of reappraisal come at little self-control effort [36]. When initiated early in the emotion generation process, reappraisal consumes little if any self control resources; when initiated late, reinterpreting the emotional contents (“online regulation”, see [3]) results in expenditures of self control resources. Another possibility is that decreasing emotions via reappraisal requires less cognitive resources than does increasing emotions [13]. So, the nuanced differences between the ostensibly same emotion regulation strategies and their resource-consuming qualities need further study.

In the current study, reappraisal group showed reduced sadness as compared to control group, whereas the ERN amplitude did not differ between reappraisal group and control group. This result seems to contradict previous findings that higher sadness related to larger ERN amplitude [37], [38]. Research on the relationship between affective sate and ERN is somewhat controversy. Changing affect using emotion-inducing pictures seemed to change ERN [38], [39], whereas inducing emotion via derogatory feedback might [37] or might not [9] enhance ERN amplitude. Therefore, future studies examining the influence of affective state and emotion changes on error detection and other performance monitoring processes are needed. One possible limitation of the present study is that we could not dissociate the influence of emotion versus regulating emotion on error detection, which most emotion regulation studies also have [13], [14], [15]. Future studies with participants regulating other emotions are needed to test of generalizability of our results.

In sum, the current study compared two different emotion regulation strategies' effect on experienced emotions and subsequent cognitive control. Based on the emotion outcome and the neural signal of performance monitoring data, reappraisal is an effective and cost free emotion regulation strategy, and suppression is an ineffective and costly emotion regulation strategy. Considering its null effect on sadness and its destructive effect on error detection, inappropriate emotion regulation (such as suppression) is worse than no emotion regulation.
